# In War and in the Future

**Published:** 1919-09-06

**Authors:** 


### THE ROYAL NAVAL MEDICAL SERVICE.

### In War and in the Future.

During the armistice period the medical organisation of the senior service came in for some perhaps unnecessarily severe criticisms from those who had done much of their war work in its ranks. In this short article it is the writer's object, after experience of service both ashore and afloat, neither to criticise nor defend, but to sketch simply an outline of the R.N.M.S. and the duties which fell to its officers during the years of active warfare, and then to trace some of the probable results which may be expected to follow this tremendous circumstantial stimulus and which may be of outstanding importance to those young doctors who may favour permanent service with our sea forces.

Throughout the war the naval surgeon found himself in one of three situations—a rough classification it is true, but sufficient at the outset. First came the shore establishment, namely hospitals, depôt ships, barracks, air stations, etc.; second, the ships which owing to their age or circumstances rarely if ever went into major action, but had to withstand the strain and monotony of endless patrolling; and third, the first-class modern fighting vessels, which alone had the good fortune to meet the Germans in the open seas. For the doctor the particular class had, quite apart from personal considerations, a very definite influence on the relative importance of his various duties and the type of ideal towards which he must strive. If we attempt briefly to describe these main subdivisions, some insight may be obtained to his duties and his life in general.

Except under emergency conditions the shore establishments provided him both with the most actual medical work and the opportunity carefully to perform it. Those fortunate or expert enough to be appointed to naval hospitals were in the position of acting in magnificently equipped establishments, with almost every afternoon sufficiently free from routine duties to allow of considerable detailed observation of patients and unusual conditions. The very "routine" itself, cumbersome though it at first appeared to the newcomer, was of material assistance in investigation of case series and the like. At no time was there so great a pressure as in the Army of urgent casualties to be dealt with by the naval units and the resulting opportunity for valuable if less outstanding investigational work is demonstrated by several most useful contributions which have appeared in the *R.N. Medical Journal* during the past four years. Also in these shore appointments the newly joined surgeon-lieutenant obtained insight and early training in the very necessary machinery by which the Navy cares so wonderfully for its men whether sick or well. It was impossible to work under these conditions without fully realisng that in the service, either in war or peace, the duties of a medical officer by no means end with the diagnosis and prescribing of treatment. The all-important processes by which men, of all degrees of intelligence, are actually provided with the many things they may require, and that with maximum efficiency both to themselves and the service, form a study in themselves. It is only by a perfect grasp and sense of proportion of the principles and requirements of both the purely medical and relatively executive branches of his work that the naval doctor can achieve justifiable distinction, and it is by initial and periodic work in the shore establishments that he must be instructed and perfected towards this ideal.

Turning now to the second class—namely, high-seas patrol—we see a type of duty which was of essentially war-time character. The medical man was faced with the possibility of having to deal with almost any type of medical and surgical case without possibility of shore assistance. Considering the difficulties and necessarily makeshift equipment encountered in these older and adapted ships, there often arose situations which greatly taxed the ingenuity of the doctor, and in themselves provided almost unique experience in developing the practical rather than the theoretical side of his profession. His life afloat was often a time of unavoidable tedium, but varied by moments of intense stress and excitement in which frequently the whole ship's company were equally involved, but often only the medical staff in a cramped and improvised sick-bay. Generally speaking, work in a patrol ship consisted of continuous striving against difficulties to organise and care for the health of her crew along the lines possible in her modern and more perfectly equipped sisters, and passing to the third class, the ships of the Grand Fleet, one may conclude this summary of the work at sea, by briefly outlining the duties in a typical fighting unit.

In this war the Grand Fleet duties have, on the whole, been relatively light in the medical sense. Crews were carefully selected and the seaman from his habitual life alone is an unusually healthy person. Except for the periodic ravages of influenza, epidemic disease was largely unknown, and the remoteness of the wartime stations kept the men remarkably free from the many complaints coincident with frequent shoregoing. The inevitable accidents and sporadic cases it was always possible to send to shore establishments with not more than a few days' delay, and even following the few major actions wounded were evacuated in a relatively short time, all except emergency treatment being supplied in the big naval hospitals. Action itself provided some of the most strenuous medical work of the war, and those surgeons who came under heavy and effective enemy fire experienced a situation which will survive as a life-long memory. The "doc." saw nothing of the action, but heard and felt a great deal. Preferably his dressing station and operating theatre were arranged in an armour-protected part of the ship, providing the wounded with relative safety from direct injury, but combining this advantage with a well-nigh complete impossibility to escape should the ship founder. Aid-posts stationed in all parts of the ship sent down the wounded as they were brought in. Several decks down they were taken by aid of hoists, hammocks, chutes and until, arriving at the protected areas, they were dealt with by the surgeon and his assistants, who performed such operations as were possible and made the sufferers as comfortable as circumstances would permit, until fortune decided whether the home ports should again receive them or the ship, without their rescue being possible, take her final plunge.

In harbour and in peace there are many odd jobs frequently entrusted by custom to the medical department. Keeping of mess accounts, wine books, letter-censoring, and a host of small wardroom duties may fall to their lot, and these in practice form a welcome link with their executive *confrères*, who in the author's own experience comprise some of the finest types of British manhood one could ever wish to meet.

But, leaving the immediate past and the days of war, when the young men from the hospitals entered in numbers as surgeon probationers or surgeon lieutenants to play some part in the nation's struggle, let us briefly consider the peace-time outlook and the circumstances a man may expect to meet if he decides to take a naval medical career. To the naval, as to every other medical service, the war has given its stimulating influence. Modernisation has been carried on apace during the last few years, both in equipment and treatment of disease. A process of welding the medical methods of the times into the already proven naval organisation has reached a stage of perfection which, with the aid of hospital ships, new shore establishments and laboratories, will present to the newcomer a picture very changed from that of pre-war times. The influence of consultants, the many brilliant younger men holding temporary posts of responsibility, and the urgent needs of the hour have greatly altered and improved the purely medical and surgical sides of the service. We think that the administrative side has been and still is admirable in itself, and that the main requirement was simply scientific and technical modernisation. The experience of war has not only automatically brought this into being, but also raised both the standard of professional requirements from and possible attainments of medical men entering the naval service. On a later page we give details of entry, pay, allowances, etc., and here in conclusion we may say that to him who would see the world he lives in, meet and treat disease under a thousand varying climatic and local circumstances, and have it in his own hands to observe, record and contribute to the advance of medical science, the Royal Naval Medical Service offers excellent opportunities.

